# Perceptions of and Preferences for Telemedicine Use Since the Early Stages of the COVID-19 Pandemic: Cross-Sectional Survey of Patients and Physicians

**DOI:** 10.2196/50740

**Published:** 2023-11-07

**Authors:** Sanae Mazouri-Karker, Robin Lüchinger, Olivia Braillard, Nadia Bajwa, Sophia Achab, Patricia Hudelson, Melissa Dominicé Dao, Noelle Junod Perron

**Affiliations:** 1 E-health and Telemedicine Division Geneva University Hospitals Geneva Switzerland; 2 Unit of Development and Research in Medical Education, Faculty of Medicine, University of Geneva Geneva Switzerland; 3 Primary Care Division Geneva University Hospitals Geneva Switzerland; 4 Department of General Pediatrics at the Children’s Hospital, Geneva University Hospitals Geneva Switzerland; 5 Treatment Centre ReConnecte, Department of Psychiatry, University Hospitals of Geneva Geneva Switzerland; 6 Clinical and Sociological Research Unit, WHO Collaborating Centre for Training and Research in Mental Health Geneva Switzerland; 7 Department of Primary Care Medicine, Geneva University Hospitals Geneva Switzerland; 8 Medical Directory, Geneva University Hospitals Geneva Switzerland

**Keywords:** acceptability, acceptance, adoption, attitude, cross sectional, e-consultation, eHealth, opinion, patient, perception, perspective, physician, questionnaire, remote consultation, survey, telehealth, telemedicine, usage, video consultation

## Abstract

**Background:**

While the use of telemedicine (TLM) increased worldwide during the early phases of the COVID-19 pandemic, little is known about the use and acceptance of TLM post the COVID-19 pandemic.

**Objective:**

This study aims to evaluate patients’ and physicians’ self-reported use, preferences, and acceptability of different types of TLM after the initial phases of the COVID-19 pandemic.

**Methods:**

We conducted a cross-sectional survey among patients and physicians in Geneva, Switzerland, between September 2021 and January 2022. Patients in waiting rooms of both private and public medical centers and emergency services were invited to answer a web-based questionnaire. Physicians working in private and public settings were invited by email to answer a similar questionnaire. The questionnaires assessed participants’ sociodemographics and digital literacy; self-reported use of TLM; as well as preferences and acceptability of TLM for different clinical situations.

**Results:**

A total of 567 patients (309/567, 55% women) and 448 physicians (230/448, 51% women and 225/448, 50% in private practice) responded to the questionnaire. Patients (263/567, 46.5%) and physicians (247/448, 55.2%) generally preferred the phone over other TLM formats and considered it to be acceptable for most medical situations. Email (417/567, 73.6% and 308/448, 68.8%) was acceptable for communicating exam results, and medical certificates (327/567, 67.7% and 297/448, 66.2%) and video (302/567, 53.2% and 288/448, 64.3%) was considered acceptable for psychological support by patients and physicians, respectively. Older age was associated with lower acceptability of video for both patients and physicians (odds ratio [OR] 0.03, 95% CI 0.00-0.33 and OR 0.23, 95% CI 0.08-0.66) while previous use of video was positively associated with video acceptability (OR 3.16, 95% CI 1.84-5.43 and OR 3.34, 95% CI 2.91-5.54). Psychiatrists and hospital physicians were more likely to consider video to be acceptable (OR 10.79, 95% CI 3.96-29.30 and OR 3.97, 95% CI 2.23-7.60).

**Conclusions:**

Despite the development of video, the acceptability of video remains lower than that of the phone for most health issues or patient requests. There is a need to better define for which patients and in which medical situations video can become safe and efficient.

## Introduction

Telemedicine (TLM) designates the use of advanced communication technologies in health care settings to provide care at a distance. Remote communication can be synchronous (phone or video) or asynchronous (email or SMS text message).

A number of studies have evaluated patients’ and physicians’ satisfaction with different means of remote communication (phone, video, email, and SMS text messaging) [1-18]. However, most of these studies were conducted before the SARS-CoV-2 pandemic and in contexts where TLM was already well developed. In these studies, the advantages listed by patients were numerous and included easier, faster, and more efficient access to health care and the opportunity to include family members more easily in the consultation [9,13,15]. Disadvantages included concerns about data protection, depersonalized care [10,15], an absence of human contact [1], and the inability to carry out certain clinical investigations [17].

The COVID-19 pandemic disrupted many aspects of health care delivery and put pressure on health systems to rapidly adapt in order to respond to patients’ health care needs. In several countries, public health authorities relaxed existing regulations to promote and facilitate the use of TLM services as part of the response to this crisis [19-22]. In some countries, TLM use, especially videoconferencing, increased enormously during the initial phases of the pandemic because it reduced the exposure to COVID-19 (and other communicable diseases) of both frontline health professionals and vulnerable patients, improved triage and care pathways for COVID-19–positive patients, and helped reduce overcrowding in emergency departments [23-27]. However, while several studies have looked at TLM use during the height of the COVID-19 pandemic [28-32], little is known about TLM use beyond the initial phases of the COVID-19 pandemic and whether video consultations have become an accepted means of providing health care for patients and physicians.

While Switzerland has a relatively well-developed telehealth ecosystem, TLM is usually limited to providers such as health insurance companies offering teleconsultations before visiting a primary care physician [33,34]. During the early phases of the COVID-19 pandemic, the Swiss Medical Association encouraged the use of videoconferencing across the country and published a fact sheet to inform physicians of the technical possibilities for conducting secure TLM consultations, the legal bases governing TLM consultations and their pricing, and the risks associated with some of the most common videoconferencing tools [35]. In early 2020, the University Hospital of Geneva, Switzerland, created and disseminated a TLM-secured application initially developed for teleconsultations between hospital physicians and home care nurses. The application was made available to both institutional and private physicians to conduct secure video consultations with their patients [36].

The aim of this study was to explore patients’ and physicians’ self-reported use of and preferences for TLM in Geneva, Switzerland, after the COVID-19 pandemic restrictions were lifted. We were particularly interested in their views regarding the acceptability of different TLM formats, including video consultations, for specific clinical situations. Understanding postpandemic perceptions and practices will help to better inform future developments in telehealth care.

## Methods

### Design and Setting

We conducted a cross-sectional study in Geneva, Switzerland, between September 2021 and January 2022.

### Participant Recruitment

Patients were recruited by research assistants in the waiting areas of 3 walk-in clinics (2 private and 1 at a public hospital), 4 primary care medical centers (3 private and 1 at a public hospital), and 1 public mental health outpatient medical center. All French-, Spanish-, Portuguese-, or English-speaking patients aged 18 years or older were invited to complete the web-based survey. Patients could complete the survey immediately on a tablet provided by a research assistant, on their smartphone, or at their convenience on their phones through a QR code posted on the Geneva University Hospitals website. Informed and written consent was obtained after explaining the study objectives. Patients received a CHF 10 (US $11) voucher for their participation.

To recruit physicians, email addresses were obtained from the Geneva University Hospital administration and the Geneva Medical Association. Email invitations were sent to all physicians (residents, chief residents, attendings, and heads of services) working in outpatient settings at the Geneva University Hospitals (n=2248) and all physicians working in private practices in Geneva (n=2715). Reminder emails were sent 2-4 weeks after the initial invitation.

Patients were recruited during September and October 2021, and physicians during December 2021 and January 2022.

### Questionnaire Development

We constructed 2 versions (for patients and for physicians) of a 27-item, web-based questionnaire. Both questionnaires contained items that assessed the respondent’s sociodemographic characteristics, digital literacy, perceived changes in use of TLM since the COVID-19 pandemic (more often to less often to no use), general preferences for 5 different communication formats (face-to-face, phone, video, email, and SMS text message; ranking 1-5), and opinions regarding the acceptability of different TLM formats for specific clinical situations. In addition, physicians were asked about facilitators and barriers regarding phone and video (open-ended responses; Multimedia Appendix 1), while patients were asked about their perceptions of confidentiality and data security of phone and video consultations (Likert scale 1-5; 1=totally disagree and 5=totally agree).

In order to explore respondents’ opinions about the acceptability of different formats of TLM for different clinical situations, we defined five common health care situations experienced by patients (and physicians): (1) information transmission: receiving (or providing) test results; (2) medical advice: receiving (or providing) medical advice; (3) clinical follow-up: monitoring a chronic problem; (4) psychological support: receiving (or providing) support for mental health and psychosocial well-being; and (5) patient requests: requesting (or responding to a request for) a medical certificate or other document. For each situation, we asked respondents to indicate acceptable formats of TLM communication (yes or no).

The questionnaires were piloted with 10 patients and 10 primary-care physicians for clarity and comprehension and subsequently modified. The patient questionnaire was translated by native speakers into English, Portuguese, and Spanish (the 3 most common languages in Geneva, after French). The translated questionnaires were then back-translated by different native speakers to verify congruence.

We used the web-based survey software Qualtrics (Qualtrics) to create and administer both questionnaires [23]. Questionnaires contained a brief explanation of the study and a request for informed consent (a check box).

### Analysis

Descriptive statistics were produced for patients’ and physicians’ preferences and opinions about the acceptability of different TLM formats. Patient and physician differences in opinions were analyzed using the chi-square test. Differences in patients’ opinions about phone versus video with regard to trust, confidentiality, perceived understanding of the health problem by their physician, and quality of care were analyzed using the McNemar test. A *P* value of ≤.05 was considered statistically significant for both tests. We conducted multivariate analyses using logistic regression to identify patients’ and physicians’ characteristics associated with the acceptability of phone and video in specific clinical contexts.

All statistical analyses were conducted using Stata Statistical Software (version 15; StataCorp) [37].

The responses to open-ended questions about physicians’ perceptions of barriers and facilitators to video and phone consultations were read by 4 investigators (PH, SMK, MDD, and NJP). Categories were identified, and a list of codes was developed, which PH then used to code all comments. Coding was checked by SMK, MDD, and NJP, and any discrepancy was resolved through discussion.

### Ethical Considerations

The study was granted a waiver from ethical approval by the ethical committee of the Canton of Geneva (Article 2 of the Swiss Federal Act on Research involving Human Beings) because we collected no personal health information.

## Results

### Participant Characteristics

Responses were obtained from 567 patients and 448 physicians (Tables 1 and 2). Patient response rate was 60% (567/940; reasons for refusal were not recorded). The response rate was 10% (225/2248) for hospital physicians and 8.5% (223/2715) for physicians in private practices.

Two-thirds of patients were aged 45 years or younger, and a majority had attended high school or vocational training. Less than a third of patients consulted more than 3-4 times per year. Most patients had internet access and used a computer or smartphone daily. Regarding TLM, they mainly reported using phone calls and, to a lesser extent, emails (Table 3).

**Table 1:** Patients’ demographics (n=567).

Sociodemographic data			Patients, n (%)
**Age (years)**			
	<30	188 (33.1)	
	30-44	181 (31.9)	
	45-64	145 (25.6)	
	≥65	53 (9.3)	
**Gender**			
	Female	309 (54.5)	
	Male	254 (44.8)	
	Other	4 (0.7)	
**Place of questionnaire fulfillment**			
	Emergency settings	383 (67.5)	
	Medical centers	119 (21)	
	Social media	65 (11.5)	
**Questionnaire filled in**			
	French	505 (89.1)	
	English	33 (5.8)	
	Portuguese	19 (3.3)	
	Spanish	10 (1.8)	
**Working time**			
	Full-time	243 (42.9)	
	Part-time	131 (23.1)	
	No work	145 (25.6)	
	Retired	48 (8.5)	
**Education**			
	No school	7 (1.2)	
	Compulsory school	88 (15.5)	
	Vocational training	155 (27.3)	
	High school	259 (45.7)	
	Other	58 (10.2)	
**Perceived health status**			
	Excellent	89 (15.7)	
	Very good	195 (34.4)	
	Good	226 (39.9)	
	Average	44 (7.8)	
	Poor	13 (2.3)	
**Frequency of medical consultation**			
	1 time/year	127 (22.4)	
	2 times/year	117 (20.6)	
	3-4 times/year	157 (27.7)	
	5-12 times/year	107 (18.9)	
	>12 times/year	59 (10.4)	
**Established care with a regular physician**			
	Yes	470 (82.9)	
**Duration of physician-patient relationship**			
	<6 months	35 (7.5)	
	6 months-2 years	116 (24.7)	
	2-5 years	198 (23)	
	>5 years	210 (44.8)	

**Table 2:** Physicians’ demographics (n=448).

Sociodemographic data		Physicians, n (%)
**Age (years)**		
	<40	135 (30.1)
	40-50	137 30.6)
	>50	176 (39.3)
**Gender**		
	Female	230 (51.3)
	Male	216 (48.2)
	Other	2 (0.4)
**Place of practice**		
	Urban	411 (91.7)
	Suburban	34 (7.6)
	Rural	3 (0.7)
**Type of practice**		
	Private practice	225 (50.2)
	Solo	94 (21)
	2-4 physicians	80 (17.9)
	Medical center	51 (11.4)
	Hospital or institution	223 (49.8)
**Working time**		
	Full-time	241 (53.8)
	Part-time	207 (46.2)
**Working experience**		
	<5 years	47 (10.5)
	5-10 years	72 (16.1)
	>10 years	329 (73.4)
**Specialty**		
	General internal medicine	179 (40)
	Psychiatry	97 (21.6)
	Other	172 (38.4)

**Table 3:** Participants’ access to and use of connected devices.

Digital use data		Patients, n (%)		Physicians, n (%)
**Access to the internet**				
	Yes	550 (97)	446 (99.6)	
	No	14 (2.5)	1 (0.2)	
	Does not know	3 (0.5)	1 (0.2)	
**Frequency of internet use**				
	Everyday	520 (92.7)	430 (97.3)	
	A few times a week	23 (4.1)	10 (2.3)	
	A few times a month	12 (2.1)	1 (0.2)	
	Less than 1 time per month	4 (0.7)	0 (0)	
	Never	2 (0.3)	1 (0.2)	
**Presence of connected tools**				
	Computer	457 (80.6)	444 (99.1)	
	Smartphone	510 (89.9)	417 (93.1)	
	Pad	246 (43.4)	244 (54.5)	
	None	5 (0.9)	12 (2.7)	
	Other	22 (3.9)	3 (0.7)	
**Usage of connected devices**				
	Phone calls	476 (83.9)	393 (87.7)	
	Video calls	329 (58)	293 (65.4)	
	Emails	484 (85.4)	434 (96.9)	
	Instant messaging	481 (84.8)	372 (83)	
	Work	349 (61.5)	389 (86.8)	
	Information seeking	407 (71.8)	429 (95.8)	
	Games	223 (39.3)	295 (65.8)	
	Other	0 (0)	0 (0)	
**Consultation format ever used**				
	Telephone	411 (72.5)	N/A^a^	
	Email	193 (33)	N/A	
	Video	39 (6.9)	N/A	
	Instant messaging	73 (12.9)	N/A	
	None	97 (16.6)	N/A	
	Other	22 (3.9)	N/A	

^a^N/A: not applicable.

Most physicians were general internists and psychiatrists, worked in an urban setting, and had been working for more than 10 years (Table 2). Half of them worked in private practices. Most physicians reported using phone and email more often than video in their everyday life (Table 3).

### Changes in TLM Use Since the COVID-19 Pandemic

**Figure 1:**
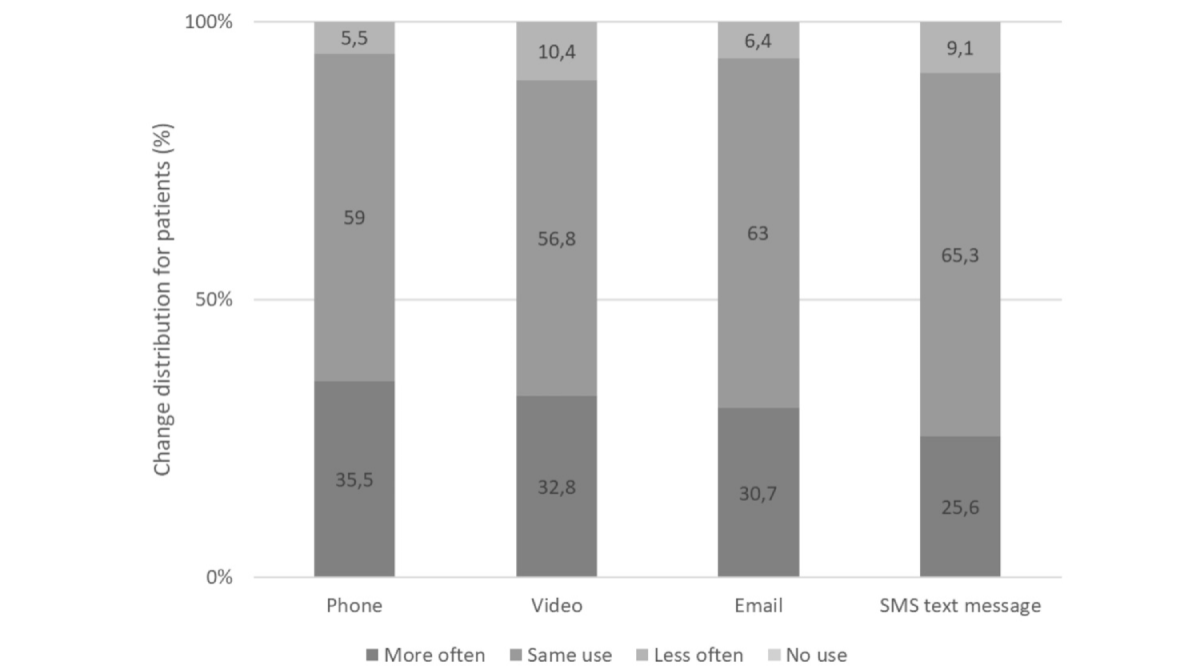
Patients' self-reported changes in telemedicine communication since the COVID-19 crisis.

**Figure 2:**
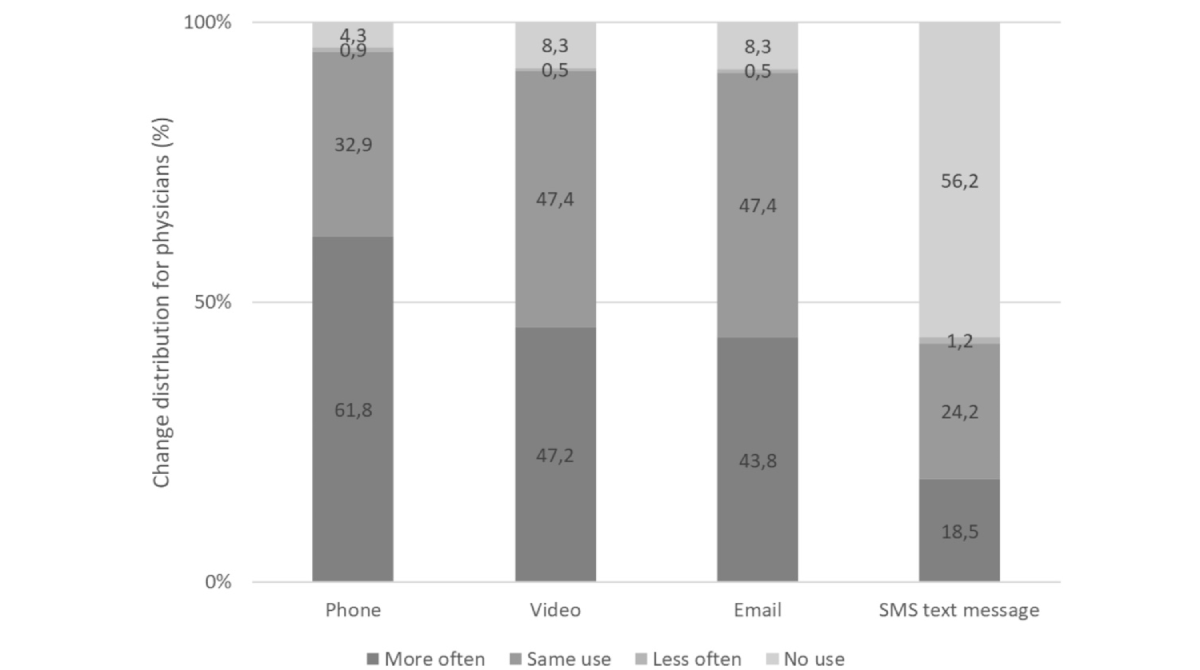
Physicians' self-reported changes in telemedicine communication since the COVID-19 crisis.

About a third of patients and half of the physicians reported using the phone, email, and video more frequently in their everyday lives since the COVID-19 pandemic (Figures 1 and 2).

### Preferences for Future Communication and Acceptability of TLM Formats for Common Health Issues

**Figure 3:**
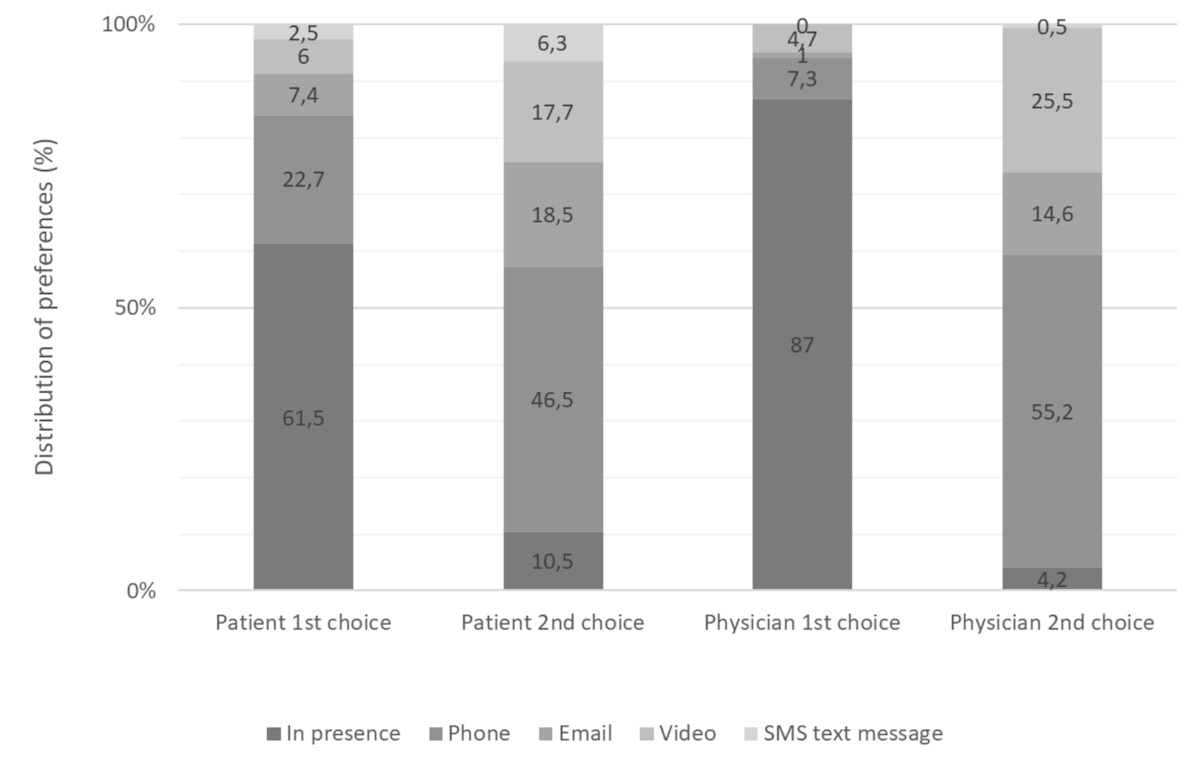
Telemedicine preferences for future consultations (ranking presentation of the 2 first choices in percentage).

**Figure 4:**
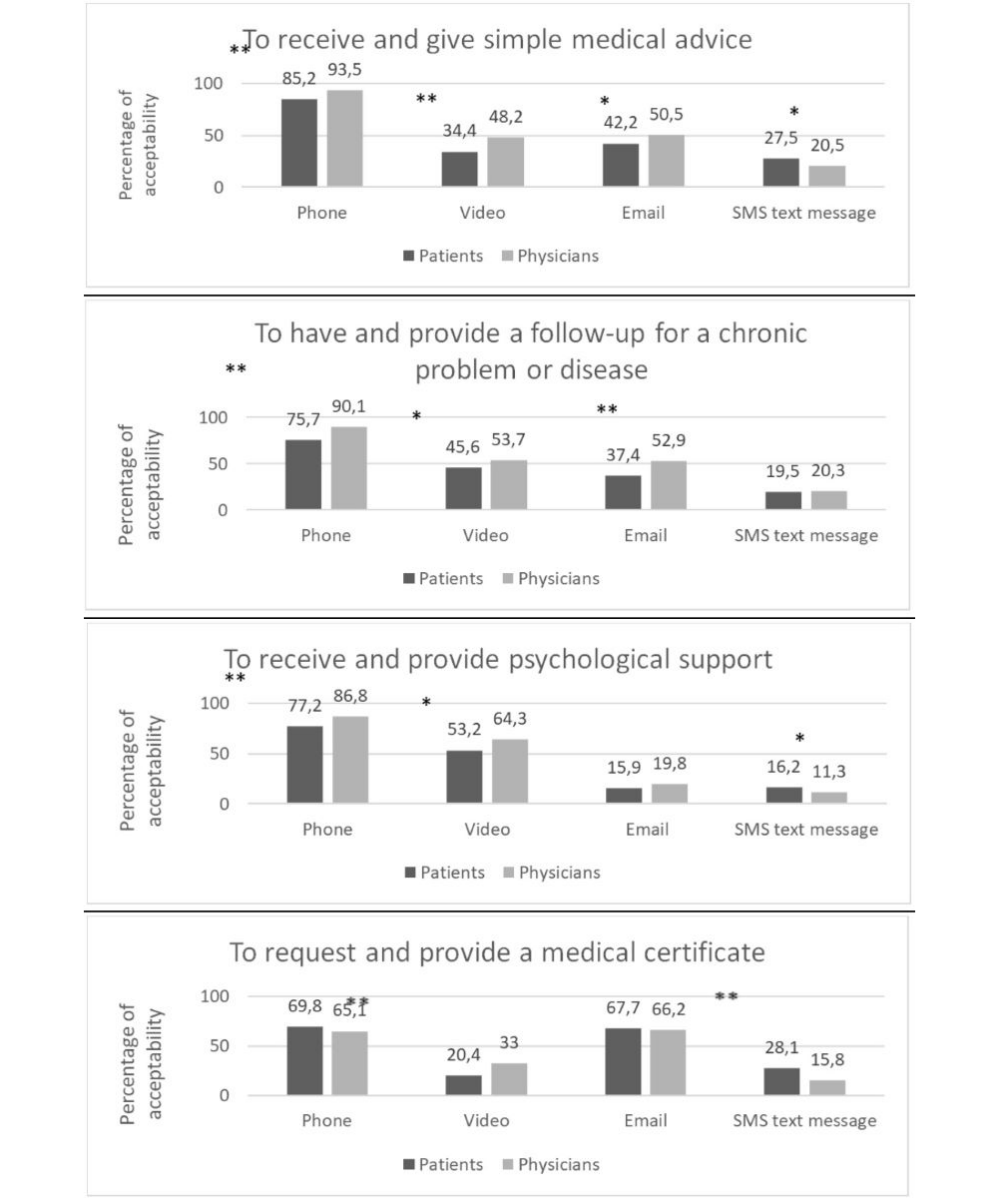
Perceptions of telemedicine acceptability for specific clinical situations (%). *P<.05 with a chi-square test, **P>.001 with a chi-square test.

Both physicians and patients ranked phone calls as the preferred TLM format after the COVID-19 pandemic (Figure 3) and considered it to be acceptable for most medical situations (Figure 4). Email was considered acceptable by both doctors and patients when requesting or providing documents. Video consultations were considered acceptable by both patients and physicians for psychological support.

### Patients’ and Physicians’ Perceptions of Phone and Video Consultations

Patients trusted phone more than video for security and confidentiality reasons (n=411, 72.5% vs n=339, 59.8%; *P*<.001) and felt better able to communicate their needs through phone than by video (n=381, 67.2% vs n=336, 59.8%; *P*<.001). There were no differences between phone and video in terms of a physician’s ability to understand their health problem (n=320, 56.4% vs n=311, 54.9%; *P*=.41) or the quality of care provided (n=180, 31.7% vs n=189, 33.3%; *P*=.25).

Physicians thought both phone and video could facilitate access to care, contribute to time efficiency, and be used for consultations not requiring a physical examination (Table 4). Perceived barriers included a negative impact on the relationship and communication, technical difficulties, inadequate financial compensation, and unsuitability for patients who lack digital literacy with connected devices (eg, older people).

**Table 4:** Written comments of physicians regarding facilitators and barriers of phone and video.

Category			Examples of physicians’ quotes
**Facilitators**			
	Expands access to care	For certain situations where travel is difficult, this allows for a consultation COVID-19, illnesses that make it impossible to come to the practice (for the patient as well as for myself)	
	Time efficiency	Teleconsultation could be an ideal way to avoid wasting work hours	
	Usefulness for specific clinical contexts	Use it for consultations that do not require a physical examination (prescription, medical certificate, laboratory results, psychology, etc)	
**Barriers**			
	Impact on relationships and communication	Loss of quality of the human relationship through the filter of a machine	
	Inability to conduct a clinical examination	Inability to examine patients through video Clinical examination is essential most of the time	
	Technical difficulties	Poor sound and image quality	
	Limited compensation	Very limited reimbursement for telemedicine	
	Unsuitable situations	Not all elderly patients have access to the technology needed to perform video consultation My patients are elderly and do not master smartphones or computers For some patients, coming to the clinic is part of the therapeutic process (getting dressed or getting out of the house)	

### Patient and Physician Factors Associated With Acceptance of Phone and Video Consultations

Private use and previous use of video with physicians were associated with patients’ acceptance of video consultations for most clinical situations, while frequent consultations were associated with patients’ acceptance of phone consultations (Tables 5 and 6). For both patients and physicians, older age was negatively associated with acceptance of video. Physicians working in hospitals and psychiatrists and physicians with previous use of video in general were more likely to accept video than others.

**Table 5:** Patient-related factors associated with their acceptance of phone and video.

Patient-related factors				OR^a^ (95% CI)
**Receiving and transmitting examination results**				
	**Phone**			
		Survey filled at an emergency center	0.55 (0.31-0.90)	
	**Videoconference**			
		Medical follow-up (2-5 years)	0.32 (0.11-0.90)	
		Use of video calls in general	3.16 (1.84-5.43)	
		Previous use of video calls with physician	3.28 (1.40-7.70)	
**Receiving and giving advice for a simple medical problem**				
	**Videoconference**			
		>65 years old	0.03 (0.00-0.33)	
		Female	0.63 (0.40-0.99)	
		Use of video calls in general	2.03 (1.27-3.25)	
**Receiving and providing psychological support**				
	**Phone**			
		Poor health status	0.23 (0.070.78)	
		Consultations (>12 times/year)	6.97 (2.12-22.92)	
		Survey filled at an emergency center	2.34 (1.27-4.32)	
	**Videoconference**			
		>65 years old	0.04 (0.00-0.39)	
		Very good health status	2.18 (1.10-4.35)	
		Use of video calls in general	2.00 (1.26-3.15)	
		Previous use of video calls with physician	4.79 (1.34-17.13)	
**Requesting and providing a work or sickness certificate**				
	**Phone**			
		Consultations (>12 times/year)	3.91 (1.35-11.30)	
	**Videoconference**			
		Compulsory school	0.23 (0.07-0.75)	
		Medical follow-up (2-5 years)	0.34 (0.11-0.99)	
		Use of video calls in general	4.00 (1.66-9.66)	

^a^OR: odds ratio.

**Table 6:** Physician-related factors associated with their acceptance of phone and video.

Physician-related factors				OR^a^ (95% CI)
**Receiving and transmitting exam results**				
	**Phone**			
		Psychiatry discipline	0.30 (0.09-0.99)	
	**Videoconference**			
		Hospital physician	3.29 (1.90-5.71)	
		Use of video calls in general	3.34 (2.91-5.54)	
		Psychiatry discipline	2.69 (1.38-5.22)	
**Receiving and giving advice for a simple medical problem**				
	**Videoconference**			
		Hospital physician	3.97 (2.23-7.06)	
		Use of video calls in general	3.08 (1.84-5.16)	
**Receiving and providing psychological support**				
	**Phone**			
		Female	0.47 (0.23-0.96)	
		Hospital physician	0.21 (0.09-0.50)	
	**Videoconference**			
		>50 years old	0.23 (0.08-0.66)	
		>10 years of working experience	3.84 (1.05-11.57)	
		Hospital physician	4.49 (2.30-8.77)	
		Use of video calls in general	3.61 (2.07-6.29)	
		Psychiatric discipline	10.79 (3.96-29.38)	
**Requesting and providing a work or sickness certificate**				
	**Phone**			
		>50 years old	0.39 (0.17-0.91)	
		Hospital physician	0.51 (0.29-0.90)	
	**Videoconference**			
		>50 years old	0.23 (0.08-0.66)	
		>10 years of working experience	3.84 (1.05-11.57)	
		Hospital physician	4.49 (2.30-8.77)	
		Use of video calls in general	3.61 (2.07-6.29)	
		Psychiatric discipline	10.79 (3.96-29.38)	

^a^OR: odds ratio.

## Discussion

### Overview

Our survey results suggest that since the COVID-19 pandemic, patients and physicians have used remote means of communication more often. Both stated a preference for the phone over other TLM formats, and patients expressed more trust in the phone than video for confidentiality and safety reasons. However, emails, SMS text messages, and video consultations were all considered acceptable, depending on the clinical situation or health request. Previous use of video calls was a key factor in patient and physician acceptance of video consultations.

The COVID-19 pandemic and perceived health risk were important factors for TLM and video acceptance [26,28] and boosted its use in several countries even after the end of the lockdown period [29]. Our data show that the COVID-19 pandemic changed physicians’ practices regarding phone and video as well as email beyond the first phases of the pandemic. However, similarly to other studies [30], both patients and physicians still preferred face-to-face consultations, with phone consultations being their second choice.

Synchronous TLM formats such as phone and video consultations differ from face-to-face consultations in that they deal with a lower number of problems and contain less exchange of information [31]. In addition, phone consultations do not allow access to visual examination and nonverbal communication [32]. However, video calls result in fewer medication errors, greater diagnostic accuracy, and improved decision-making accuracy when compared to phone consultation. Teleconsultations by phone or video appear to offer an effective alternative to face-to-face consultations in terms of patient satisfaction and costs in primary care and mental health services [38,39].

To our knowledge, little is known about patients’ preferences or acceptance of TLM regarding different health issues. Our findings showed that patients and physicians found phone consultations to be highly acceptable for most health issues (advice, follow-up, psychological support, and certificates) and trusted phone more than video for security and confidentiality reasons. Savira et al [30] showed in a discrete choice experiment that patients had no preference between face-to-face, phone, or video regarding issues such as repeat prescription or surgical follow-up but felt that TLM was not appropriate for more complex or sensitive issues or when a physical examination was required. However, another study showed that patients tended to consider phone to remain the preferred synchronous TLM format because of video limitations related to technology and privacy concerns [40]. In this study, both physicians and patients were more willing to accept video for psychological support. This finding is in line with several studies reporting that patients with mental health issues also consider video acceptable when they have a preestablished relationship with their therapist, when their issues are less complex, or when they encounter barriers to accessing their therapist’s office [39]. Similarly, psychotherapists also tend to value video consultations as a potential means to improve access to mental health care [41]. Such popularity may be explained by the fact that psychiatric follow-ups consist of long-term engagements with the same therapist for narrative clinical work rather than physical exams and show low levels of variation from a consultation sequence to another compared with consultations with primary care physicians.

While acceptance of SMS text messaging remains low for all clinical situations displayed in this study, emails are largely accepted for simple medical advice and the provision of documents. These findings are somewhat similar to previous studies showing that patients tended to use emails for clinical (medical and treatment) rather than administrative requests [42-44].

Several “pre-COVID-19” studies showed that patient factors associated with acceptance of TLM, particularly video, were regular use of video for calls, previous experience of video with their physician, younger age, and being male [45-51]. Patients also tended to be more accepting of phone and video for routine health issues, particularly when there was a preexisting relationship with their physician [18,30,31,52].

In this study, physicians’ acceptance of video was associated with working in a hospital or as a psychiatrist. It is possible that hospital physicians felt more institutional pressure to use videoconferencing or were more rapidly equipped to conduct such consultations. Another explanation may be that they felt they were expected to improve access to specialized care for some patients and to improve collaboration with family physicians during the COVID-19 pandemic [41]. Psychiatrists’ inclination to adopt video consultations to permit better access to mental care for their patients has already been reported [6].

Most studies assessing factors that could affect the intention of physicians found that physicians who perceived integrating telehealth in their clinical practice as part of their professional and social responsibilities and felt comfortable using TLM expressed a stronger intention to use this technology [3,46,49,53]. Additional predicting factors of intention to use TLM were the potential to reduce cost and a positive perception of medical information security and confidentiality [54,55]. Factors such as the development of user-friendly video platforms with improved interoperability between digital systems and the involvement of telehealth coordinators, together with adequate reimbursement of digital services, may also accelerate such a shift to TLM [34,56-59].

### Limitations

The response rate was low for both physicians and patients. Participating physicians may have been more interested in and familiar with TLM than nonparticipants. Patient nonresponders may have included patients with low digital literacy (eg, older people). We also excluded patients who were unable to answer the questionnaire in 1 of the 4 languages available. This may have influenced our results, as both age and language ability are factors negatively associated with acceptance of TLM. Furthermore, we recruited patients mainly at emergency centers, which may have added an additional bias since patients consulting emergency centers may not be representative of the general patient population. Finally, this study was conducted in a single, primarily urban Swiss canton. Patients and physicians in other cantons or more rural areas may have different perceptions of the usefulness and acceptability of TLM.

Despite these limitations, our results offer some insight into the factors influencing postpandemic TLM-related practices and the opinions of patients and physicians.

### Conclusion

Although Swiss physicians modified their TLM practices with higher self-reported use of TLM since the COVID-19 pandemic, use and acceptability of video remain rather low, except for mental health support [21], despite the relaxation of existing regulations and policies to promote and facilitate the use of video as well as improved access to secure videoconference platforms.

### Practice Implications

Improved interoperability between digital systems and adequate reimbursement of digital services may accelerate a shift to video if both patients and physicians obtain higher guarantees regarding confidentiality and security issues and are better informed about the criteria and health conditions allowing adequate video. Although video seems to be highly acceptable and effective for individuals with mental illness and their therapists, there is a need for further studies to better define for which patients and in which medical situations video is safe and efficient.

## Appendix

Multimedia Appendix 1 Physicians questionnaire.

## Abbreviations

OR: odds ratio
TLM: telemedicine
