# Supplementary material for: Perceptions of and Preferences for Telemedicine Use Since the Early Stages of the COVID-19 Pandemic: Cross-Sectional Survey of Patients and Physicians
Source: JMIR Hum Factors. 2023 Nov 7;10:e50740. doi: 10.2196/50740 (PMC10664018; doi:10.2196/50740)
Supplement: Multimedia Appendix 1 [file humanfactors_v10i1e50740_app1.docx]

Telemedicine: Physician Survey

Survey Flow

Block: Default question block (1 Question)

Standard: Block 1 (8 Questions)

Standard: Block 2 (5 Questions)

Standard: Block 3 (3 Questions)

Standard: Block 4 (5 Questions)

Standard: Block 5 (2 Questions)

| Page Break |  |
| --- | --- |

Start of Block: Default question block

Q1
You are invited to participate in a survey conducted by a team from the University Hospitals of Geneva (HUG) and the University of Geneva. It has been submitted to the Commission Cantonale d'Ethique de la Recherche en Suisse (exemption granted).


The purpose of this study is to evaluate your perceptions and preferences regarding the modes of communication in telemedicine, i.e., for health care or advice delivered by telephone, video, email or instant messaging. The results of this research will help to propose recommendations for best practices.


Your participation consists of completing a questionnaire that should only take 10 minutes. You are free to accept or decline to participate in the survey. You decision and withdraw from the project at any time without having to justify your decision. project without having to justify yourself.

The data collected for research purposes are anonymous. The results may be used in scientific publications. The confidentiality of the data will be ensured, and your name or any your name or any information that could identify you will not appear anywhere. All persons involved in the study are bound by confidentiality. confidentiality.

You You may contact the principal investigator of the survey at any time sanae.mazouri@hcuge.ch / +41795530154

 By agreeing to participate in this study, you consent to the use of your data for research purposes.

- I wish to participate in the study. (1)
- I do not wish to participate in the study. (2)

Skip To: End of Survey If you are invited to participate in a survey conducted by a team from the University Hospitals of... = I do not wish to participate in the survey.

End of Block: Default question block

Start of Block: Block 1

| 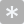 |
| --- |

Q2 What is your year of birth (e.g. 1987)

________________________________________________________________

Q3 What is your gender?

- Male (1)
- Female (2)
- Other (3)

Q4 What is your medical specialty?

- Anesthesia (1)
- Angiology (8)
- Allergology-immunology (9)
- Cardiology (10)
- Surgery (specify) (11) __________________________________________________
- Dermatology (2)
- Endocrinology / diabetology (3)
- Gastroenterology (4)
- Medical genetics (5)
- Gyneco-obstetrics (7)
- Hematology (12)
- Infectiology (13)
- Occupational medicine (14)
- General Internal Medicine (15)
- Physical medicine and rehabilitation (16)
- Tropical medicine (17)
- Nephrology (18)
- Neurology (19)
- Oncology (20)
- Ophthalmology (21)
- ENT (22)
- Pediatrics (23)
- Pharmacology (24)
- Pneumology (25)
- Adult psychiatry (26)
- Child and adolescent psychiatry (27)
- Radiology (28)
- Radio-oncology (29)
- Other (6) __________________________________________________

Q5 What is your primary township of practice?

- AG (1)
- AI (2)
- AR (3)
- BE (4)
- BL (5)
- BS (6)
- EN (7)
- GE (8)
- GL (9)
- GR (10)
- JU (11)
- LU (12)
- NE (13)
- NW (14)
- OW (15)
- SG (16)
- SH (17)
- SO (18)
- SZ (19)
- TG (20)
- IT (21)
- UR (22)
- VD (23)
- VS (24)
- ZH (25)
- ZG (26)

Q6 What is your primary place of practice?

- Urban (1)
- Semi-urban (2)
- Rural (3)

Q7 What is your primary facility type?

- Individual (1)
- 2 - 4 physicians (2)
- Medical Center (3)
- Hospital / Institution (4)

Q8 What is your activity rate?

|  | 0 | 10 | 20 | 30 | 40 | 50 | 60 | 70 | 80 | 90 | 100 |
| --- | --- | --- | --- | --- | --- | --- | --- | --- | --- | --- | --- |

| Activity rate in percent () | 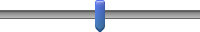 |
| --- | --- |

Q9 How many years of experience have you had since obtaining your federal degree or equivalent?

- < 5 years (1)
- 5-10 years (2)
- > 10 years (3)

End of Block: Block 1

Start of Block: Block 2

Q10 Do you have access to the Internet at home?

- Yes (1)
- No (2)
- I don't know (3)

Q11 What connected tools do you have?

- Computer (1)
- Smartphone (2)
- Tablet (3)
- Other (4) __________________________________________________
- None (5)

Display This Question:

If What connected tools do you have? = None

Q12 How often do you use these connected tools?

- Every day (1)
- A few times a week (2)
- A few times a month (3)
- Less than once a month (4)
- Never (5)

Display This Question:

If What connected tools do you have? = None

Q13 How do you use your connected tools?

- Making calls (1)
- Make video calls (2)
- Send - receive emails (3)
- Send -receive instant messages (4)
- Working (5)
- Search for information (7)
- Gaming / social networks / online shopping (6)

Q14 Does your practice/office have computer software for managing patient records?

- Yes (1)
- No (2)
- I don't know (3)

End of Block: Block 2

Start of Block: Block 3

Q15 In your interactions with your patients, please indicate the means of communication other than traditional face-to-face that you feel are acceptable for the following situations (multiple choices possible):

|  | Telephone (1) | Mail (2) | Video (3) | Instant messaging (4) |
| --- | --- | --- | --- | --- |
| Communication of test results (laboratory, radiology) (1) |  |  |  |  |
| Discussion of self-measured values at home (blood pressure, sugar level, pain intensity, ...) (2) |  |  |  |  |
| Follow-up for a chronic disease/problem (high blood pressure, diabetes, osteoarthritis, etc.) (3) |  |  |  |  |
| Follow-up of an urgent illness / problem (urinary infection, cold, gastroenteritis, back blockage, contusion, etc. ....) (4) |  |  |  |  |
| Simple medical advice (guidance on what to do for a new health problem) (5) |  |  |  |  |
| Psychological support (6) |  |  |  |  |
| Request for a voucher for a consultation with another health professional (specialist, dietician, physiotherapist, etc.) (7) |  |  |  |  |
| Application or extension of work/sickness certificate (8) |  |  |  |  |
| Prescription renewal (9) |  |  |  |  |

Q16 Since the COVID crisis, what changes in the ways you communicate with your patients have you experienced?

|  | More often (1) | Same thing (2) | Less often (3) | No use (4) |
| --- | --- | --- | --- | --- |
| Telephone (1) |  |  |  |  |
| Mail (2) |  |  |  |  |
| Video (3) |  |  |  |  |
| Instant messaging (6) |  |  |  |  |

Q17 What communication methods would you like to use for future consultations? (drag and drop)

______ Phone (1)

______ Mail (2)

______ Video (3)

______ Instant messaging (4)

______ Presential (5)

End of Block: Block 3

Start of Block: Block 4

Q18 In your opinion, what are/would be the barriers to the adoption of video consultation in your daily practice?

________________________________________________________________

________________________________________________________________

________________________________________________________________

________________________________________________________________

________________________________________________________________

Q19 In your opinion, what are/would be the barriers to the adoption of telephone consultation in your daily practice?

________________________________________________________________

________________________________________________________________

________________________________________________________________

________________________________________________________________

________________________________________________________________

Q20 What conditions do you think would encourage you to use telemedicine in your daily practice?

________________________________________________________________

________________________________________________________________

________________________________________________________________

________________________________________________________________

________________________________________________________________

| 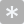 |
| --- |

Q21 In your experience, what are the benefits of remote consultation with your patients? Please select up to 5 statements that you feel are most important from the following:

- This saves my patients a trip. (1)
- This saves my patients time (3)
- This makes it easier for my patients' family caregivers to participate. (6)
- This allows for a shorter consultation than in person. (7)
- This allows for a less expensive consultation than in person. (19)
- This saves my patients the trouble of arranging childcare. (8)
- This saves my patients from having to take time off work. (9)
- This allows my patients to get a quicker response or medical advice. (10)
- This allows for continued follow-up while traveling or living abroad. (11)
- This allows for closer medical follow-up than in person. (12)
- This saves my patients from going to an emergency room or another physician. (13)
- It allows my patients to talk about more things (15).
- This allows me to be more flexible on appointment times than in person. (16)
- This allows my patients to stay at home and feel less anxious about going to the doctor (less white coat effect). (17)
- ⊗I see no benefit to teleconsultation. (20)

| 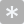 |
| --- |

Q23 In your opinion, what are the main disadvantages of communicating with your patients remotely? Please choose up to 5 statements that you feel are most important from the following:

- This does not guarantee confidentiality, as my patients are not always alone in their homes or workplaces. (1)
- This does not always guarantee data security depending on the platform used. (2)
- This exposes technical problems (equipment, connection, sound and/or image quality). (3)
- This sometimes requires downloading a specific software or application (4)
- This does not always lend itself to the medical situation. (5)
- This does not allow for close medical follow-up (6)
- This does not allow for a physical examination (7)
- This does not allow for good quality communication. (8)
- This does not allow my patients to ask all their questions because the time is limited. (9)
- This results in less participation from my patients. (10)
- This is associated with a less warm, less friendly contact. (11)
- This does not allow for physical contact (different from physical examination). (12)
- The timing of the teleconsultation is sometimes poor. (13)
- This requires special skills to communicate via video. (14)
- ⊗I see no disadvantage to teleconsultation. (15)

End of Block: Block 4

Start of Block: Block 5

Q22 Are you willing to be contacted by phone for an interview about telemedicine? If yes, please provide your name and phone number. (e.g. Mr. Muster 0221234567)

- No (1)
- Yes (2) __________________________________________________

Q24 You have reached the end of the questionnaire. This space is reserved for you to share experiences or comments related to telemedicine.

________________________________________________________________

________________________________________________________________

________________________________________________________________

________________________________________________________________

________________________________________________________________

End of Block: Block 5
